# Structural background of intraspecific color polymorphism and the driver of geographic patterns in a shining leaf chafer

**DOI:** 10.1186/s12983-025-00571-5

**Published:** 2025-08-04

**Authors:** Yuanyuan Lu, Alexander Kovalev, Lulu Li, Chuchu Li, Xinyi Zhu, Min He, Xingke Yang, Ming Bai, Stanislav N. Gorb

**Affiliations:** 1 State Key Laboratory of Animal Biodiversity Conservation and Integrated Pest Management, Institute of Zoology, Chinese Academy of Sciences, Beijing, 100101 China; 2 Department of Functional Morphology and Biomechanics, Zoological Institute, University of Kiel, 24118 Kiel, Germany; 3 University of Chinese Academy of Sciences, Beijing, 101408 China; 4 Academy of Plateau Science and Sustainability, Qinghai Normal University, Xining, 810016, China

**Keywords:** Color, Polymorphism, Melanin, Structural color, Rutelinae, Thermoregulation

## Abstract

**Background:**

The phenomenon of color polymorphism has been extensively documented in a range of animal species. A series of hypotheses have been proposed to explain potential functions of color variations in diverse habitats. However, the generation of color is an intricate physical, chemical and biological process. In this instance, the attempts to explain the distribution patterns and their potential causes lacking structural background of color formation, are likely to be misguiding.

**Results:**

Here we studied the distribution pattern of color phenotypes in the beetle *Popillia mutans* (Insecta: Coleoptera: Rutelinae). Three phenotypes (blue, green and red) are distributed in a not mutually exclusive manner, with the blue phenotype tending to be more prevalent in the cooler northern area, seemingly following Bogert's rule, and the others mainly in the warmer southern area. Subsequent analysis demonstrated that this type of distribution correlates with the environmental factor of temperature. Based on the optical, mechanical and chemical characteristics of the cuticle, we found that this species represents a special case in which melanin layering causes structural coloration, whereas pigmentation plays a primary role in red phenotype and physical coloration is the dominant factor in blue and green phenotypes. However, the structural alterations within the cuticle have no influence on its mechanical properties, different from previously suggested. We have also shown that the blue phenotype exhibits a slightly faster heating rate than the other phenotypes facilitating their adaptation to lower-temperature regions.

**Conclusion:**

Our results elucidate the structural background of color and identify the possible natural selection factor from an evolutionary standpoint. This aids in understanding species formation, as well as prospective dynamic distribution of the phenotypes under the pressure of climate change.

**Supplementary Information:**

The online version contains supplementary material available at 10.1186/s12983-025-00571-5.

## Introduction

Color polymorphism, when multiple discrete color phenotypes occur within a single species, has been extensively documented in various animals, including arthropods, amphibians, birds and mammals [1–5]. The abundance of species in insects allows for examining color phenotypes and their geographic distribution patterns in multiple lineages [6, 7]. The reported cases have revealed that the distribution pattern of color polymorphism in insects could be related to latitude, longitude or altitude, and the corresponding environmental factors, such as temperature, precipitation, and solar radiation may be responsible for this [3, 6, 8, 9]. This type of color variation provides an ideal opportunity to explore the role of potential environmental stresses in the species formation and divergence, as well as their prospective dynamic distribution affected by climate change [9].

Traditionally, color polymorphism has been regarded as an evolutionary adaptation enabling species to occupy a wider range of habitats or niches [1, 10, 11]. A series of hypotheses have been proposed to explain potential functions of color variations in diverse habitats, including camouflage, thermoregulation, mechanical resistance and UV protection [10, 12, 13]. However, the generation of color is an intricate physical, chemical and biological process. In addition, in some instances, color is merely a secondary consequence of microstructures that fulfill other specific physiological functions [14–16]. In this instance, any attempts to explain the distribution patterns and potential causes lacking structural background of color formation, are likely to be misguiding [17].

The existing research on insect coloration mechanisms has made strong progress in the course of last decade. The majority of studies have revealed two mainly mechanisms of the colors: (i) the pigment-based colors, such as the red and black observed in ladybirds [18] and (ii) physical coloration, including multilayer interference structural blue and green colors in jewel beetles and butterflies [19, 20], scattering white colors in scarab beetles and fruit flies [21, 22], diffraction grating-based iridescent luster in beetles and butterflies [16, 23], photonic crystals [23], and the combination of these principles. Given the interdisciplinary nature of related studies, which encompass biology, physics and chemistry, the majority of the research studies are focused on the physical principles of color formation or on single functional aspect of the color [24, 25]. The current state of research, comprising isolated studies, may result in an incomplete understanding of the color generation, their potential function and ecological significance. Comprehensive studies that integrate the morphological background, physical mechanisms and functional ecology of color polymorphism are relatively scarce [13] and therefore there is a clear need for further investigation in this research area.

This research employs an integrated methodology to investigate the occurrence of color polymorphism in the diurnal scarab beetle, *Popillia mutans* (Insecta: Coleoptera: Rutelinae), and to ascertain possible underlying causes. The genus *Popillia* comprises more than 300 species, many of which are considered agricultural and forestry pests (eg. *Popillia japonica*) [26]. The adults of *Popillia* are diurnal and active during the warm seasons and spend most of their time feeding and mating on the surface of host plants. Of particular significance is the observation that many widespread species exhibit color polymorphism. Among them, *P*. *mutans* is an ideal model to address the mechanism and function of polymorphism, given its relatively homogeneous coloration in certain phenotypes and its displayed distribution pattern among color phenotypes. *P. mutans* with several color phenotypes and widely spread in the lowlands of most provinces in China and adjacent countries (North Korea, South Korea, Vietnam, and India) (Fig. S1 in Additional file 1) [27, 28]. Herein, first, the distribution pattern of color phenotypes of *P*. *mutans* was described, and an attempt was made, based on a series of statistical analyses, to identify the geographical pattern and their correlated environmental variables. Second, an analysis of the morphological, optical, mechanical, and chemical characteristics of the elytral cuticle has revealed the mechanism of color polymorphism as being based on layering of pigment melanin involved in the differentiation of the three main color phenotypes. Third, a series of experiments was designed based on the species’ habits and the previously selected environmental variables to prove functional hypothesis about difference in mechanical properties and in thermoregulation capabilities of the cuticle in different color morphs. Our findings presented here provide some support for the hypothesis about the thermoregulation function, which is probably due to the difference in the melanin amount in different morphs. The results provide deeper insight into potential environmental stresses that may have been occurred during the divergence of color phenotypes, which might be applied for elucidating the relationship between the dynamics of species color pattern distribution and the effect of the climate change on it.

## Materials and methods

### Specimen distribution data

**Fig. 1:**
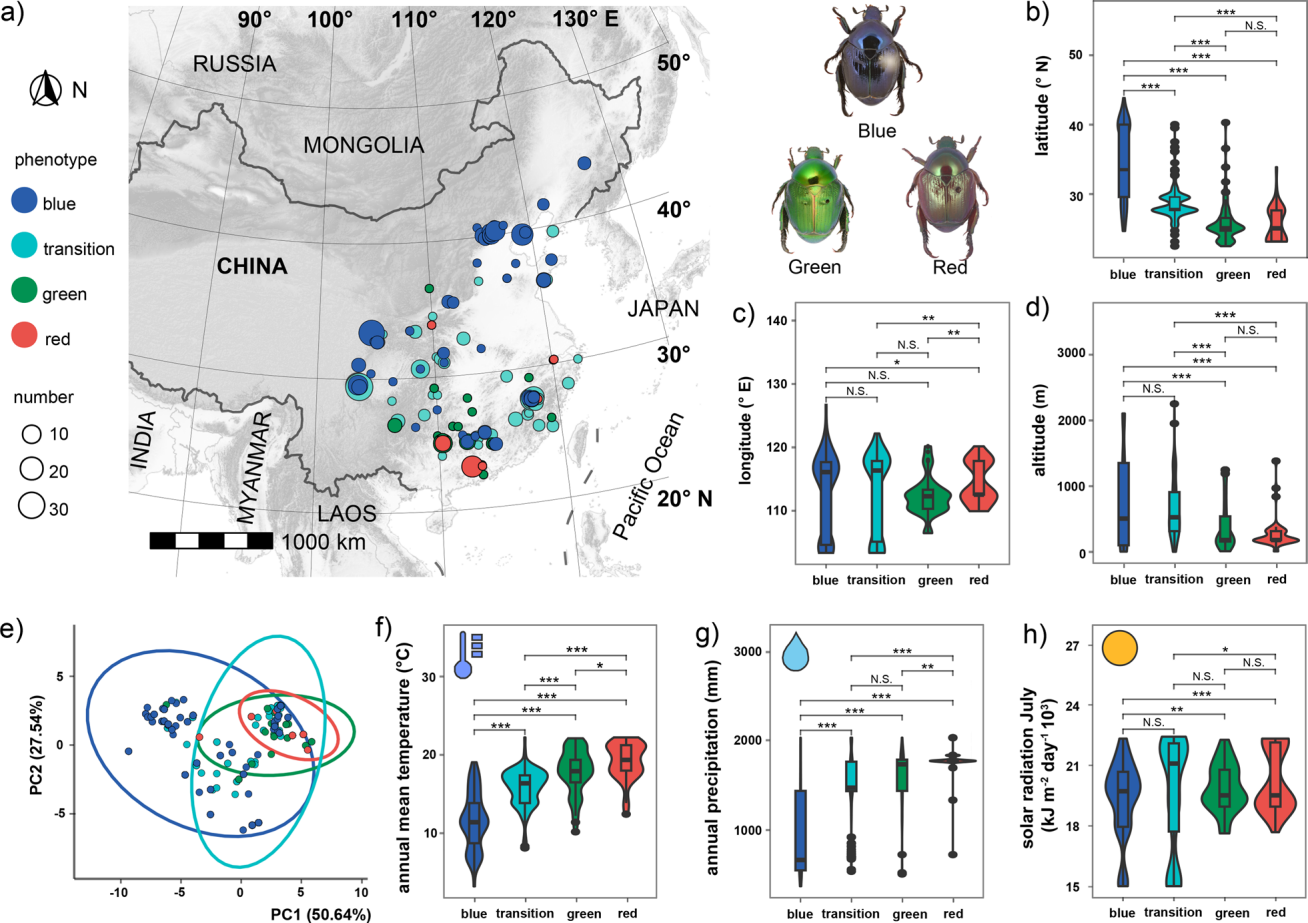
Distribution of the color morphs in *Popillia mutans.* **a** Map showing distribution of *P. mutans* colors phenotypes. Transition means mixed occurrence of blue and green color phenotypes. **b**–**d** Differences of collection sites in latitude (**b**), longitude (**c**) and altitude (**d**) among color phenotypes. **e** The PCA plot showing the distribution of specimens with different color phenotypes based on the environmental variables (Bio_1 to 19 and Srad_1 to 12) along principal component axes (PC1 and PC2). **f**–**h** Differences in one environmental factor among different color phenotypes: annual average temperature (**f**), annual precipitation (**g**), Solar radiation in July (**h**). **b**–**d**, **f**–**h** Significant differences evaluated by the *t *test. Asterisks indicate the level of significance (N.S. *p* > 0.05, *p < 0.05, **p < 0.01 and ***p < 0.001)

The color patterns of 544 adults of *Popillia mutans* from China were examined in this study (Additional file 2). All specimens were deposited in the entomological collection of the Institute of Zoology, Chinese Academy of Sciences (IZCAS), Beijing. The distribution map of specimen's richness at each locality is based on the specimen's collective coordinates in the Lambert Azimuthal equal area projection (PROJ: +proj=laea+lat_0+lon_0=104) using the R package "sf", "ggspatial", and "ggplot2" (Fig. 1a).

To analyze correlation between the color phenotype distribution and environmental factors, such variables as longitude, latitude, elevation (SRTM Dataset v.4.1) and other 43 variables were selected (Table S1 in Additional file 1): 19 bioclimatic variables including temperature and precipitation (Bio_1 to 19) (MERRAclim v.2.0) [29], 12 months mean temperature (Tavg_1 to 12) and 12 months solar radiation (Srad_1 to 12) (Table S2 in Additional file 1). The 43 environmental variables plus elevation with 10 min (~ 340 km^2^) spatial resolutions were downloaded from WorldClim (http://www.worldclim.com/version2), then the R package "raster" was used to extract these variables from each location. Principal component analysis (PCA) was performed to compare the differences in environmental variables (Bio_1 to Bio_19, Tavg_1 to 12) among the color phenotypes using the "princomp" function in R package "stats" (Fig. 1e). To ensure the environmental variable matrix comparable in PCA, it was first centered and scaled using the "scale" function. The violin plots (Fig. 1b–d, f–h, Figs. S2–S5 in Additional file 1) were made by "ggplot2", and the *p*-values between each two color phenotypes were calculated by two-tails *t*-test using the R package "ggsignif".

In order to select relatively independent environmental variables, the Pearson correlation coefficients of the cross-correlations among 43 bioclimatic variables were calculated using the function "cor()" and R package "corrplot". The environmental variables, which Pearson correlation coefficient was ≥ 0.8 (|r|≥ 0.8) were omitted (Additional file 2). First, Bio_1 and Bio_12, as basic factors were considered, while the other variables with higher Pearson correlation coefficient (|r|≥ 0.8) were omitted. Then more variables with higher Perarson correlation coefficient by rank from Bio_1 to Bio_19 were omitted.

### Optical microscopy observation

For proving the presence of melanin in the beetle cuticle, the elytra were dissected from dry specimens, and then bleached in the 30% solution of hydrogen peroxide (H_2_O_2_) for 48 h.

**Fig. 2:**
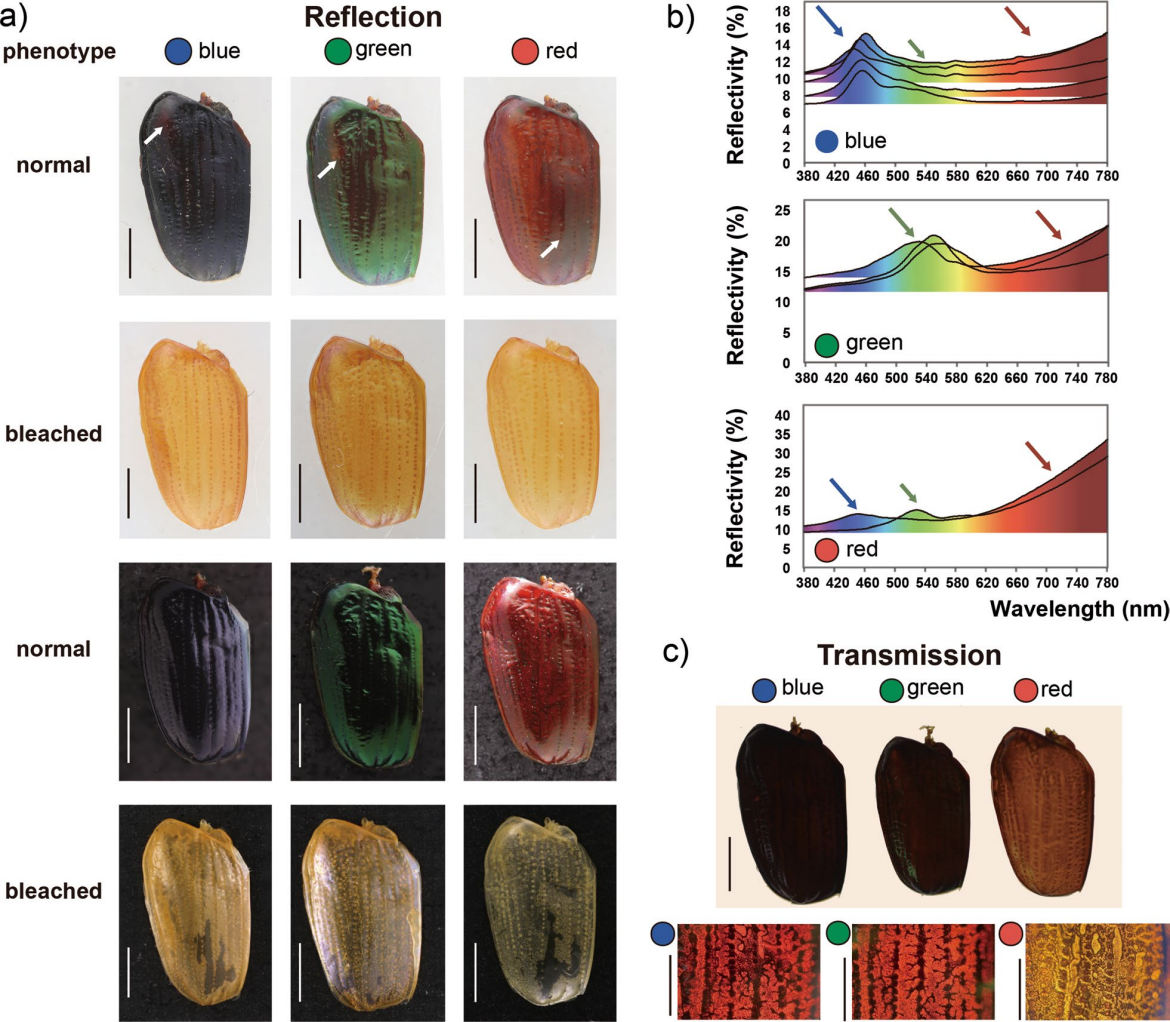
Optical effect of three color phenotypes (blue, green, and red). **a** Elytra of three color phenotypes (from left to right: blue, green, and red) before bleaching and after bleaching on different backgrounds under top light illumination, white arrows in blue and green phenotypes show the red areas, white arrow in the red phenotype shows the green area, scale bars = 2 mm. **b** Reflection spectra of the elytra in the visible spectral range (wavelengths 380–780 nm), the arrows indicated peaks. **c** The elytra of three color phenotypes (from left to right: blue, green, and red) under the optical light microscope (brightfield illumination). Upper: dorsal side of elytra; Lower: partial area of elytra in dorsal view magnification about twice that of upper elytra, scale bar (upper) = 2 mm, scale bars (lower) = 1 mm

The Olympus SZ61 stereomicroscope was used to conduct observations and dissections. Before and after bleaching, the elytra were photographed by Canon 5D digital camera combined with a Canon MP-E 65 mm f/2.8 1-5× Macro Lens (Fig. 2a, c). The illumination source was used from the top or bottom, respectively. The initial images were then stacked in Helicon Focus v.7.0.2 and processed in Adobe Photoshop 2021.

**Fig. 3:**
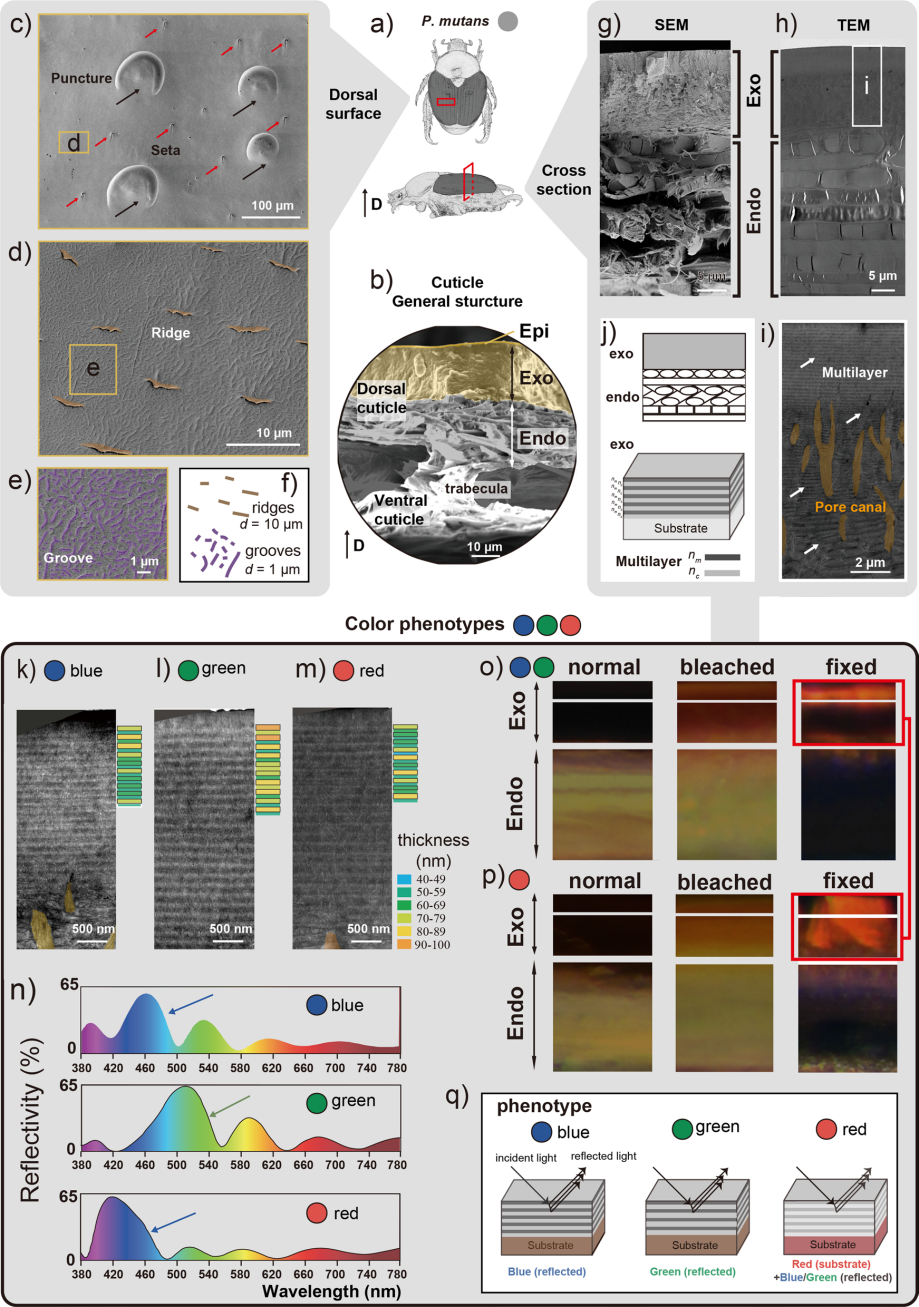
Microstructural characterization of the cuticle in *P. mutans*. **a** Dorsal and lateral view of the beetle, D indicates the dorsal direction. **b** SEM image of the cross-section of the beetle elytra showing the general structure of the cuticle, from upper surface to below: epicuticle (Epi: orange region), exocuticle (Exo: yellow region) and endocuticle (Endo: grey region). **c**–**e** SEM image of the dorsal surface of elytra at different magnifications. Black arrows in (**c**) indicate punctures. Red arrows in (**c**) indicate setae. Ridges in (**e**) are colored yellow. Micro grooves highlighted purple are shown in the inset (**e**). **f** Schematic of ridges and grooves in the dorsal surface of the cuticle, *d*: distance. **g** SEM image of the cross-section showing the exo- and endocuticle. **h** TEM image showing the exo- and endocuticle, **i** exocuticle, white arrows indicate the multilayers, yellow highlighted region shows pore canals within exocuticles. **j** Modelling of the cuticle structure, upper: exo- and endocuticle model, below: Multilayer model in the exocuticle, *n*_*m*:_ refractive index of dark layer, *n*_*c*_: refractive index of pale layer. **k**–**m** External section of exocuticle in blue phenotype (**k**), green phenotype (**l**), red phenotype (**m**); the right part shows the first 20 sublayers’ thickness. **n** Modelled reflectance spectra of the multilayer system based on the first 20 exocuticle sublayers (**k**–**m**), the arrows indicated peaks in the visible spectral range. **o**–**p** autofluorescence images of cross-sections from normal, bleached, and chemically fixed elytra sections, **o** Exocuticle and endocuticle of blue and green phenotypes, **p** Exocuticle of the red phenotype. **q** Schematic representation of the probable coloration structural mechanism of the three color phenotypes of the *Popillia mutans*

The elytra were sectioned into pieces (about 20 µm) using a razor blade and then placed on a glass slide, mounted in glycerin and covered with a cover slip for further fluorescence microscopy observation (Fig. 3o, p, normal and bleached).

### Optical reflection curves

Diffuse reflectivity of elytra in the visible light range of wavelengths (380–780 nm) was measured using a spectrometer (SolidSpec-3700, Shimadzu, Japan) (Fig. 2b). The Barium Sulfate (BaSO_4_) plate was taken as a white standard for diffuse reflectance (100% reflectance).

### Scanning electron microscopy observation

The elytra were dissected from the preserved specimens, washed twice with 75% ethanol using an ultrasonic cleaner (SB-5200DTD, Scientz, Ningbo, China), with each step lasting for 20 s, gradually dehydrated in 80%, 85%, 90%, and 95% ethanol for 20 min at each concentration. This was followed by two 20-min long periods in 99.9% ethanol. After this, elytra were placed into a clean Petri dish and stored in a 40 °C electric thermostatic drying oven (GZX-GF101-2-BS-II/H, Hengzi, Shanghai, China) for 12 h until all samples were dry. The dried samples were mounted on aluminum stubs with double-sided copper sticky tape, coated Platinum for 10 mA in 55 s (thickness ~ 5 nm) using a high-resolution sputter coater (E-1045, Hitachi, Tokyo, Japan). A scanning electron microscope (SU8010, Hitachi, Tokyo, Japan) was used to observe the samples and take photos at accelerating voltage of 3 kV (Fig. 3b–e, g). In total, six specimens were examined.

### Transmission electron microscopy observation

The samples of elytra were fixed with 2.5% (vol/vol) glutaraldehyde with phosphate buffer (PB) (0.1 M, pH 7.4), washed four times in PB. Then samples were first immersed in 1% (wt/vol) OsO_4_ and 1.5% (wt/vol) potassium ferricyanide aqueous solution at 4 °C for 1 h. After washing, they were incubated in filtered 1% thiocarbohydrazide (TCH) aqueous solution (Sigma-Aldrich) at room temperature for 30 min, 1% unbuffered OsO4 aqueous solution at 4 °C for 1 h and 1% uranyl acetate (UA) aqueous solution at room temperature for 2 h. Then samples were dehydrated in ascending alcohol series (30, 50, 70, 80, 90, 99.9, 99.9%, 10 min each, at 4 °C) and finally in 99.9% acetone (2 × 10 min). Samples were infiltrated in graded mixtures (8:1, 5:1, 3:1, 1:1, 1:3, 1:5) of acetone and SPI-PON812 resin (21 ml SPI-PON812, 13 ml DDSA and 11 ml NMA, 1.5% BDMA), then in pure resin. Finally, samples were embedded and polymerized for 12 h at 45 °C, 48 h at 60 °C. The ultrathin sections (70 nm thick) were sectioned with ultramicrotome (Leica, EM UC6, Germany) and examined in a transmission electron microscope (FEI, Tecnai Spirit, Japan) at 120 kV (Fig. 3h, i, k–m).

At the same time, the embedded elytra were sectioned into pieces (20 µm thick) using microtome (RM2265, Leica, Vienna, Austria) and then mounted in glycerin on a glass slide and covered with a cover slip for further fluorescence microscope observation (Fig. 3o, p, fixed).

### Simulation of reflection spectra

The theoretical reflectance spectra of the outer exocuticle of beetle elytra were calculated in OpenFilters software [30], considering multilayer structure of the cuticle (Fig. 3n). The number of layers and their thickness, as well as the values of their complex refractive indices (RI), were used to model spectra of the normal reflection. The thickness of layers was measured from TEM images using ImageJ software (Wayne Rasband, National Institutes of Health, USA). The refractive index values of cuticular layers, used in this study, were taken from the literature [31, 32], considering the contrast difference to recognize the melanized and non-melanized layers. Pale layers were considered to have chitin as its main component, hence, *n*_*c*_ = 1.58, and dark layers were considered to be melanized, thus, *n*_*m*_ = 1.80 [31, 32].

### Fluorescence microscope observation

To visualize the autofluorescence of exocuticle and endocuticle of elytra sections (~ 20 µm thickness), they were observed in three spectral bands: blue band (excitation 340–380 nm, emission 420 nm), green band (excitation 512–546 nm, emission 600–640 nm) and red band (excitation 710–775 nm, emission 810–890 nm) using fluorescence microscope Axioplan (Zeiss, Jena, Germany) equipped with a Sony Power HAD; XBO75W and HBO 100W lamps as a light source (Fig. 3o, p).

### Mechanical characterization using nanoindentation

**Fig. 4:**
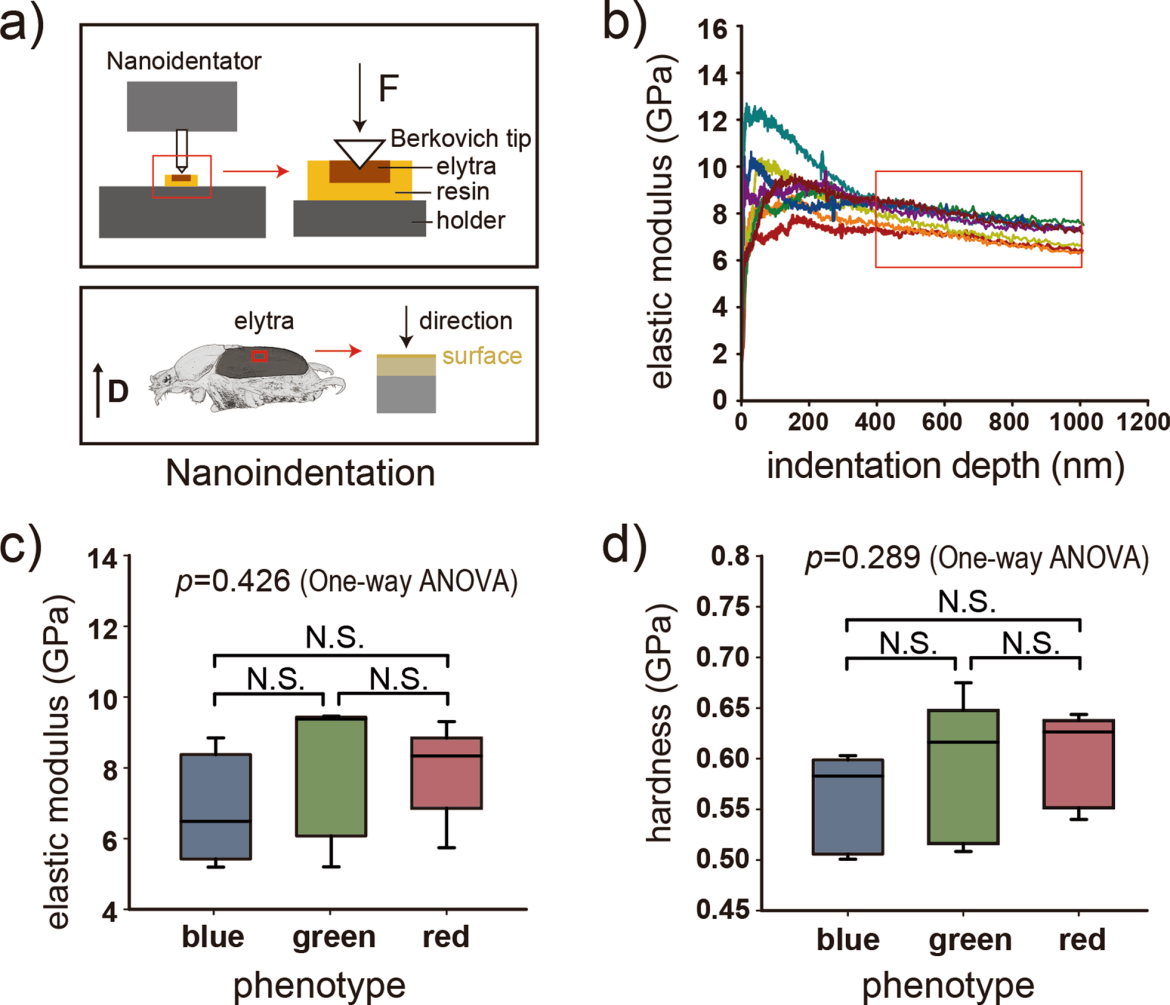
Mechanical properties of the elytral cuticle. **a** Schematic of mechanical characterization of elytral cuticle using nanoindentation. **b** Elasticity modulus as a function of the indentation depth obtained from the continuous stiffness measurement (CSM) (eight indents per specimen), the red rectangle indicates the depth range used for average elasticity modulus calculation. **c**–**d** Elasticity moduli (**c**) and hardness of the elytra (**d**) in the three color phenotypes studied. Significant differences between two phenotypes evaluated by the *t* test. Asterisks indicate the level of significance (N.S. *p* > 0.05)

The elytra were separated from the air-dried adult *P*. *mutans* beetles. They were embedded into the Spurr epoxy resin [33] and mounted on cylinder aluminum sample holders (Fig. 4a) and kept in the thermostat (U15, Memmert, Schwabach, Germany) for 24 h at 70 °C, for the epoxy resin polymerization. Subsequently, the specimens were polished using a polishing machine (Minitech 233, Presi GmbH, Hagen, Germany). Suspensions of aluminum oxide with a decreasing grain size of 12 µm, 3 µm, 1 µm and 0.3 µm were applied during the polishing process. Water was used as cooling medium. Each solution was sonicated for 15 min to ensure homogeneous mixing.

The specimens were indented using a SA2 Nanoindenter (MTS Nano Instruments, Oak Ridge, TN, USA) equipped with a Berkovich diamond tip. The elasticity modulus and hardness of the specimens were measured using the continuous stiffness measurement (CSM) technique. Nanoindentations were performed normal to the surface (Fig. 4a). In total, five (N) specimens per color phenotype were measured while 6 to 14 (n) indents were performed per specimen (Additional file 4). The distance between adjacent indents was set to > 50 μm to avoid interference between consecutive measurements. For all indentations, maximum indentation depth, strain rate, harmonic displacement and harmonic frequency were 1.0 μm, 0.05 s^−1^, 1.0 nm and 75 Hz, respectively. Poisson’s ratio of specimens was assumed as 0.3 [34–36]. The *p*-values between each two color phenotypes were calculated by two-tails *t*-test.

### Thermal characterization of samples using infrared camera

**Fig. 5:**
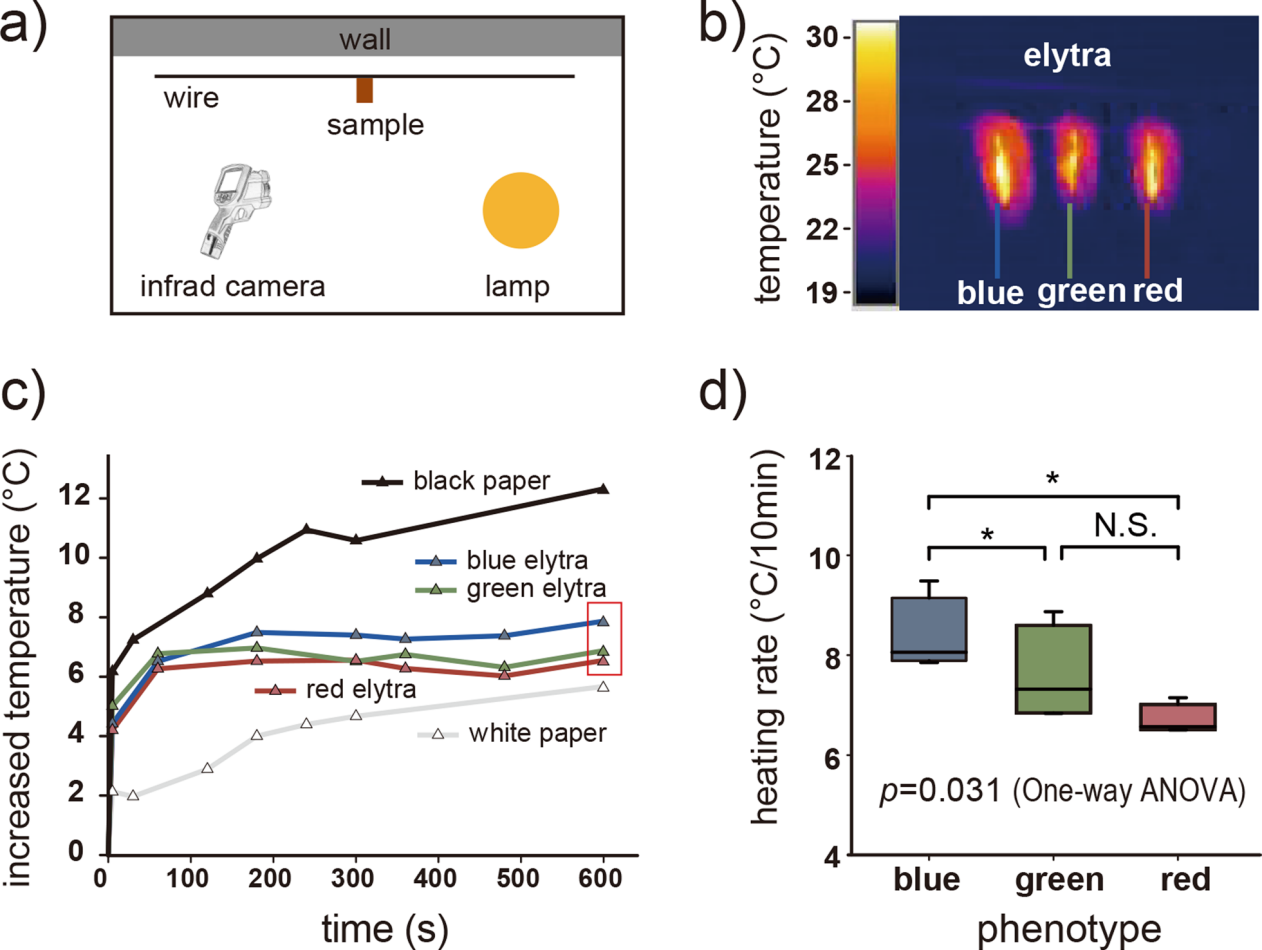
Thermoregulation ability of elytra in three phenotypes studied. **a** Experimental setup. **b** Infrared image showing the temperature distribution on the elytral surface. **c** Temperature time dependence of the blue, green and red elytra, black and white papers during continues illumination with a heat source. Temperature increases after 10 min of illumination, marked with a red rectangle, was used to calculate the heating rate. **d** The heating rate of the elytra in the three phenotypes studied. Significant differences between two phenotypes evaluated by the paired *t* test. Asterisks indicate the level of significance (N.S. *p* > 0.05, *p < 0.05)

The dorsal surface is the major area of beetles exposed to solar radiation. Therefore, we focused on the elytra, which make up the majority of the dorsal area. An 80 W lamp was used as a heat source. The temperature distribution on elytra surface was recorded with infrared camera IC085LV (TROTEC, Heinsberg, Germany) within 10 min. after lamp was switched on (Fig. 5). Elytra of all three color phenotypes, white paper and black paper were hung out at randomized positions. The experiments with three color elytra were repeated four times (n = 4) with different individuals, as contrast group, white paper and black paper were recorded once (Additional file 5). The *p*-values between each two color phenotypes were calculated by two-tails paired *t* test.

### Statistical analysis

SigmaPlot 12.0 (Systat Software Inc., San José, CA, USA) was used to perform One Way Analyses of Variance (ANOVA) statistical analysis. The data were tested for normality with Shapiro–Wilk test. The normally distributed data for different color phenotypes were compared using One Way ANOVA. Alternatively, Kruskal–Wallis One Way ANOVA on ranks was used. The significant differences between two phenotypes were calculated by *t*-test, details see the related *Method* part.

## Results

### Color polymorphism and distribution pattern

The 544 adults of *Popillia mutans* collected from 19 provinces (22 to 42 degrees latitudes) of China display four basic color patterns: blue (n = 201), transition (blue-green, n = 246), green (n = 57), and red (n = 40) (Fig. 1a). Distribution areas of them overlap with the blue color phenotype having the largest distribution area. Distribution areas show no difference in longitude (Kruskal–Wallis ANOVA on Ranks, *H* = 5.039, *p* = 0.169), but have statistically significant differences in latitude and altitude (Kruskal–Wallis ANOVA on Ranks, both *p* < 0.001) (Fig. 1b–d, Table S2 in Additional file 1). From lower to higher latitudes, the proportion of blue color patterns gradually increases from 0 to 100%, and the proportion of red and green color patterns decreases accordingly. For example, all specimens collected from Beijing (40°N) have blue color, and those from Guangdong (23°N), 55% are red, and 45% are green (Additional file 2). The corresponding latitude analysis shows that differences between each pair of phenotypes color phenotypes are greater than would be expected by chance (*t *test, *p* < 0.001), except for between the green and red (*t *test, *p* = 0.705) (Fig. 1b). In terms of altitude, the individuals with blue and transition phenotypes are located at higher elevations than those with green and red phenotypes (Fig. 1d).

To explore the relevant environmental factors associated with color phenotypes, the environmental variables related to temperature, precipitation (Bio_1 to Bio_19) and monthly solar radiation from January to December (Srad_1 to 12) were analysed (Fig. 1e–h, Figs. S2-S5 and Table S1 in Additional file 1). From the principal component analysis result (PCA), we observed that (a) the four color phenotypes have differences in associated environmental factors; (b) there was noticeable variation between blue and other color phenotypes in PC1 and PC2; (c) the green and red color phenotypes almost overlapped with each other, and only have difference in PC1 (Fig. 1e).

From the further analysis on environmental factors, we found that the distribution areas of four color phenotypes show significant difference in the Annual mean temperature (Bio_1, Kruskal–Wallis One Way ANOVA on ranks, H = 236.868, *p* < 0.001) and Annual precipitation (Bio_12, Kruskal–Wallis One Way ANOVA on ranks, H = 183.670, *p* < 0.001). Among four color phenotypes in Bio_1, between each two colors, all tested combinations have statistical differences (Fig. 1f, Table S2 in Additional file 1). In Bio_12, only between transition and green phenotypes there were no statistical differences (Fig. 1g, Table S2 in Additional file 1). After simplified analysis, in which only relatively independent environmental variables (Pearson correlation coefficients) were kept, and considering the active months of adults from June until September, six more relatively independent environmental variables were selected: Bio_2, Bio_3, Bio_5, Bio_8, Bio_15, Bio_18, and Srad_6 to Srad_8. Among them, only Bio_5 (Max temperature of the warmest month) showed stronger relationship to the color phenotypes’ distribution (Figs. S2-S4 and Table S2 in Additional file 1, Kruskal–Wallis One Way ANOVA on ranks,* p* < 0.001, and six paired color phenotypes show significant difference, *t* test, *p* < 0.05).

The results show that the distribution pattern of color phenotypes is more related to the Bio_1 (Annual Mean Temperature) and Bio_5 (Max temperature of the warmest month). Further analysis on active monthly mean temperature (Tavg_7 and 8) has similar results as Annual mean temperature (Table S2 and Fig. S4 in Additional file 1). Although the Monthly solar radiation shows relationship with the color phenotypes distribution, the differences between the color phenotype pairs were not so clear (Fig. 1h, *t* test, *p* < 0.001 only between blue and red) (Fig. S4 in Additional file 1). Therefore, we found that the distribution of color phenotypes is mainly related to the temperature (Annual mean temperature and Active monthly mean temperatures).

In considering the fact that the blue-green transition color phenotype is closely aligned with both the blue and green phenotypes, the subsequent morphological and functional verification was conducted exclusively among the three distinct color phenotypes: blue, green and red.

### Optical properties of color phenotypes

To get the first impression about optical properties of the three main color phenotypes (blue, green and red), we made images of the elytra against white and black background. On the image with white background, the blue phenotype looks dark with slight bluish metallic blaze on the periphery of elytra and has a faint reddish spot near humeral umbone (anterior distal, Fig. 2a). The green phenotype demonstrates prominent shiny green appearance with a dark area in the middle and a faint reddish spot (anterior distal) as well as the blue phenotype. The red phenotype has dark red, bright red, and desaturated red areas. The blue and green phenotypes imaged against black background demonstrate saturated colors, while bright red areas of the red phenotype became dark red. Elytra of all studied phenotypes became transparent-yellowish after oxidation of the pigments with H_2_O_2_. The dorsal surface of the elytra bleached with H_2_O_2_ in the blue phenotype looks more matt, in comparison to more specular reflecting elytra of other two phenotypes (Fig. 2a, bottom row).

Reflection spectroscopy in general supports the observation of optical imaging (Fig. 2b, Additional file 3). So, reflection spectra in the green phenotype demonstrate a peak at 531–561 nm (the reflectivity in peak: 19.8–20.9%, n = 3), in the blue phenotype a peak is at 445–461 nm (the reflectivity in peak: 11.0–15.4%, n = 5) and a shoulder at 500–540 nm. Interestingly, both blue and green phenotypes demonstrate reflection in red 700–780 nm (the reflectivity range: 7.3–22.5%), while reflection intensity in red for the red phenotype is 19.8–33.6% (n = 2), the reflection spectra of red phenotypes have reflection peaks corresponding to that of blue and red phenotypes (the reflectivity in peak 451 nm, 14.0%; 528 nm, 15.0%).

The difference (such as amount or type) of pigments could be estimated by observing the elytra in transmitted light. According to Fig. 2c, the red phenotype has much less homogeneously distributed pigment (the elytra is semitransparent) than both blue and green phenotypes. In blue and green phenotypes, the pigment is distributed inhomogeneously: black stripes along the main axis with the 0.4 µm distance between them could be seen in the light microscope. The blue phenotypes seem a little bit darker than green.

### Elytra cuticle morphology and structural coloration

In general, the elytra of *P. mutans* is composed of a thick dorsal and a thin ventral cuticular lamination and connect with some trabeculae (costal intervals) (Fig. 3a, b). The surface of dorsal cuticle (which corresponds to the epicuticle) bears similar microstructures in all three-color phenotypes (Fig. 3c–f, Fig. S6 in Additional file 1). It is rather smooth with longitudinal trabeculae, some longitudinal lined ~ 80 µm in diameter punctures (Fig. 3c), 5–20 µm long seta. Some relative regularly spaced outgrowths with elongated ridges (length ca. 2–8 µm, distance between two ridges ca. 10 µm) (Fig. 3d, f, yellow) and dense, elongated, curved or irregular grooves length from 0.2 to 3.0 µm (Fig. 3e–f, purple) are present (distance between two grooves ca. 1 µm).

The dorsal cuticle of the elytra consists of three layers: 0.2 µm thick epicuticle, 18 µm thick exocuticle and 33 µm thick endocuticle (Fig. 3 g, h, j). Exocuticle could be further splitted into two sublayers: a 6 µm thick external sublayer, in which electron lucent (grey) and electron dark layers are repeatedly alternating (around 25 times) and a ca. 12 µm thick internal sublayer with obvious pore canals running across the multilayered structure (perpendicular to the cuticle surface) (Fig. 3i, j). Below exocuticle, the massive endocuticle is located.

The thickness of the layers was different among the three color phenotypes: the grey layers in green are thicker than those of the blue, the grey and dark layers in the red phenotype shows less distinct (Fig. 3k–m). To explore possible mechanism of the color formation, the theoretical reflectance spectra of the multilayered system in the beetle’s elytra exocuticle were simulated based on the thickness of individual layers appearing grey and dark on TEM images (Additional file 3). The position and intensity of the main peak on the reflection spectra simulated based on the measured layer thickness distribution are similar to those of the measured reflection spectra for blue and green color phenotypes (Fig. 3n). The main peaks of simulated spectra of red color phenotype are close to the secondary peak in 450 nm of the measured one (Fig. 3n, bottom). The simulated spectra for all phenotypes have no obvious peaks in red 700–780 nm.

The subsequent examination of the cuticle autofluorescence shows some specificity of the red phenotype. Before bleaching, the exocuticle of all phenotypes is black, but after bleaching it becomes red which is similar to the endocuticle. In chemical fixed samples, blue and green phenotypes show red and black regions in exocuticle, whereas red phenotypes show only red autofluorescence (Fig. 3o, p).

### Mechanical properties of the elytra

To explore the mechanical properties of color phenotypes, we measured the elasticity modulus and hardness of the elytral cuticle by nanoindentation of the elytral surface in the normal direction (Fig. 4a). Typical elasticity modulus—indentation depth curves, obtained by CSM method are shown in Fig. 4b. The elasticity modulus and hardness reported for each indent was the average value of their measurements at the indentation depth from 0.4 to 1.0 μm for five individuals and ca. 10 locations (detailed information is reported in Additional file 4). The elasticity modulus of the elytra in blue, green and red phenotypes demonstrated no statistically significant difference according to one-way ANOVA (*p* = 0.426): 6.85 ± 1.36 GPa, 8.11 ± 1.72 GPa, and 7.97 ± 1.18 GPa, as well as hardness (*p* = 0.289): 0.56 ± 0.04 GPa, 0.59 ± 0.06 GPa, and 0.6 ± 0.04 GPa, respectively (Fig. 4c, d, Additional file 4).

### Thermoregulation ability of the elytra

To further explore the thermoregulation ability of the cuticle of the color phenotypes, we measured the elytral cuticle temperature from the normal direction after 10 min long exposure to the simulated solar radiation (Fig. 5a, b, Additional file 5). The white paper and black paper were simultaneously used as control samples. The surface temperature of all samples increased (from ca. 4–7 °C) within first 10 s of infrared irradiation, then slowly became stable during next 10 min (Fig. 5c). The black paper temperature increased at more than 12 °C after 10 min of infrared irradiation, and the white paper's temperature increased in contrast at 6 °C only. The temperature of the elytral surface in blue, green and red phenotypes demonstrated statistically difference according to one-way ANOVA (*p* = 0.031), and the temperature of blue elytra (8.38 ± 0.75 °C) was about 0.8 °C higher than that of the green (7.60 ± 0.95 °C), and 1.66 °C higher than red color phenotypes (6.72 ± 0.30 °C) (*p* < 0.05).

## Discussion

The phenomenon of color polymorphism is observed in a wide range of insect groups, and based on prior research, its expression can be affected by the environmental factors such as temperature, rainfall and solar radiation, while many studies provide indirect evidence and lack of experimental studies [37, 38]. In this study, the *P*. *mutans* species is investigated, to elucidate the underlying mechanism and potential ecological function of this phenomenon. The blue, blue-green transition, green and red phenotypes of *P*. *mutans* are distributed in a not mutually exclusive manner, with the blue color phenotype located in colder regions with higher elevation compared with other color phenotypes. The multifactor environmental analysis indicated that the distribution strongly correlates with the factors of temperature. Therefore, it is reasonable to assume that the distribution pattern of the blue, blue-green transition, green and red color phenotypes is at least partly driven by environmental factors. In the case of the dung beetle, *Gymnopleurus humanus*, similar geographic patterns are observed, with the blue phenotypes distributed in the cooler regions, rarely occurring in the warmer regions, and blue, green, and cupreous co-occurring in intermediate areas [6, 39, 47]. A number of examples in birds, lizards and insects demonstrate that similar distribution patterns are common among animals, with the darker coloration occurring, where the climate is cooler, summarized as so-called Bogert's rule [40, 41]. In contrast, there is Gloger's rule claiming that in some species darker coloration occurs more often in wet and warm environments [41, 42]. Hence, the fact of coloration is a complex trait compromising various selection forces, physiological functions and evolutionary constraints [40, 43, 44].

The explanation of Bogert's rule is typically simplified and considered as warming up function, because of the strong sun light absorption by melanin [44, 45], which, to some extent is true. Our examination of the optical characteristics of the phenotypes of *P*. *mutans* studied here shows that in any case, melanin or melanin-like pigments are responsible for the structural color formation in this beetle, since after melanin oxidation the color disappears (Fig. 2a). It has been also determined that the so-called 'red' coloration may bear the light absorption properties of the melanin-like pigment in the red phenotype. Moreover, the blue and green phenotypes partially display also some portion of red reflection, which is obviously less intense than in red phenotypes, but with very similar spectral shape above 640 nm (Fig. 2b). However, the overall darkness of melanin in the red elytra is less than those in the blue and green elytra. This is demonstrated by the observation under light transmission conditions, where the red phenotype appears yellow, and the blue and green phenotypes are much darker (Fig. 2c). In addition, melanin or melanin-like pigments usually lead to the autofluorescence absorption in the insect cuticle [46–48], therefore showing dark regions in the exocuticle of all three color phenotypes (Fig. 3m, n, normal). Our results additionally prove it, because the dark color disappears when the specimens are bleached. However, after the chemical fixation treatment, the autofluorescence of the red elytra in exocuticle became distinct from that of blue and green phenotypes, while blue and green elytra largely remained dark and the red elytra became red. This is very likely that due to the specific reactions of melanins to chemical treatments, e.g. glutaraldehyde and osmium tetroxide, we can assume the presence of different kinds of melanin molecules between blue + green and red phenotypes.

The shining ‘blue’ and ‘green’ hues result from the structural multilayer reflection, based on the presence of melanized dark layers and non-melanized grey layers within exocuticle, as supported by our simulation results (Fig. 3n). It is interesting that the simulation results also indicate that the red phenotype should be blue. Potentially, the less distinct layering structure may not be sufficient to cause clear blue or green coloration to suppress the red pigment color, which is likely due to the difference of melanin. Consequently, in individuals exhibiting red phenotypes, the color is typically perceived as red, although with some additional weak blue or green reflection. Herein, we speculate that the red phenotype may have different epidermal gene expression, which could result in a change in the pigment, as found in the other species [37, 49].

The present study demonstrates the complex underlying mechanism of color polymorphism in *P. mutans*, integrating both pigmentation and physical coloration. In the red color phenotype, pigmental coloration plays a primary role, while in the blue and green phenotypes, physical coloration is the dominant mechanism (Fig. 3q). This leads us to the conclusion that, when considering color polymorphism, an examination of optical reflectivity and transmission is a crucial step in differentiating between phenotypes.

The clarifying of the underlying mechanism responsible for the observed variation in color phenotypes will facilitate the further investigation of their potential ecological function and the factors influencing their distribution patterns. Two common hypotheses for the function of color polymorphism are visual signaling and camouflage [3, 50, 51]. However, it seems unlikely that this color polymorphism between blue, green and red serves a visual signaling function in the group, given that there is no sexual dimorphism, and the variation in color polymorphism occurs in the same species and overlaps within geographical groups. Aposematic coloration are also questionable, because the colors here are not typical warning colors, like combination of yellow and black [52]. As for the function of camouflage, the reflection of shining blue and green colors here is both possible in reducing the predator’s attention [53–55], while the distribution pattern among the three colors remains unclear. Sometimes, color polymorphism is manifested as a secondary consequence of microstructures on the surface, which may have a functional role in hydrophobicity or friction [15, 56]. However, this is not the case for this species, as the structural differences in surface of cuticle between the three color phenotypes are not discernible (Fig. 3b, c).

Considering the environmental stress in its habitats, this species is active during the day and stays on the upper surface of the leaves, which is (1) exposed to predator’s direct attack and (2) probably influenced by the effect of solar radiation, we tested two other hypotheses, which are the mechanical stability and thermoregulation ability (selected environmental factor, in *Result* ‘color polymorphism and distribution pattern’).

In certain instances, color polymorphism may manifest diverse mechanical capabilities [13, 57, 58]. In the case of birds, black feathers in birds are thought to exhibit enhanced mechanical properties, presumably due to the presence of melanosomes [57], and similar in beetles, the diverse range of colors, observed in the Cetoniinae group, has been showed to be the result from structural variations within the exocuticle layer, which in turn contribute to varying mechanical properties [13]. The difference in cuticle’s mechanical capabilities which could represent the defensive ability. Our morphological results presented here demonstrate variation of cuticle components among color phenotypes of *P*. *mutans* (Figs. 2, 3). Therefore, we assume that there may be variations in the mechanical properties of cuticle among color patterns. However, the nanoindentation experiments indicate that the elasticity modulus and hardness of the color phenotypes are not significantly different (Fig. 4). This suggests that the melanin layering in the elytra of *P*. *mutans* does not correlate with the mechanical properties. Our results show that the elytra of *P*. *mutans* is highly sclerotized having the elasticity modulus around 5.5–9.8 Gpa and hardness around 0.52–0.64 GPa. This complies with the mechanical properties of other beetle elytra, such as *Trypoxylus dichotomus* and *Phloeodes diabolicus *[59]. It seems that the sclerotization level of elytral cuticle is sufficient for mechanical protection [36, 60], therefore the color differences observed here are likely do not alter the mechanical function of the cuticle [61].

Thermoregulation is another possible functional interpretation of the color polymorphism [62–66]. Our results indicate that the blue phenotype heats up faster than green (11%) and red (25%), when exposed to IR-radiation. This is very likely an advantage, when living in colder regions, where the faster heating process might help the insects to faster obtain sufficient amount of energy from solar radiation in early morning for their typica activities of flying, feeding, etc. [67, 68]. The results, therefore, provide some support to the hypothesis that color phenotypes are distributed in relation to the environmental factor of annual average temperature. The more widespread distribution of the blue phenotype and the limited range of the red one, along with their demonstrated heating speed, can be explained based on their melanin difference. It is worth noting that the factors involved in the validation of thermoregulation function are extremely complex [69, 70]. It can be observed that the green color phenotypes exhibit a lack of sufficient heating speed, which may be correlated with the distribution pattern, but differs from the attributes associated with the quantity of melanin. Further research considering more factors will help us to better understand the underlying mechanisms of thermoregulation [71, 72].

## Conclusion

Coloration mechanisms in insects have been observed and analyzed in numerous taxa, among them, shining leaf chafers (Rutelinae) is the beetle group with few recent publications [73–75]. Our findings further illustrate the complexity of color generations in the representatives of this group. This study represents a special instance, whereby pigmentation and structural coloration are both engaged in the process of color formation, in addition to which their proportions and respective roles differ in accordance with the specific color phenotype. Although there is a polymorphism in color phenotype, the structural changes within cuticle are not strong and have no influence on the mechanical properties of the body. Furthermore, the blue phenotype has a slightly faster heating rate than the green and red phenotypes, which may assist in their adaptation of this species to the wider range environments and occupy larger overall area. Our research indicated that in order to ascertain the principal natural selection factors from an evolutionary standpoint, it is first necessary to elucidate the physical or chemical processes involved in the formation of color. This will facilitate our understanding of the relationship between the distribution pattern and function of color polymorphism, and provide a theoretical basis for the study of insect diversity under climate change conditions [5, 8, 9, 37, 71, 72].

## Supplementary Information

Additional file 1

Additional file 2

Additional file 3

Additional file 4

Additional file 5

## Data Availability

The datasets supporting the conclusions of this article are included within the article and its additional files.
