# Supplementary material for: Structural background of intraspecific color polymorphism and the driver of geographic patterns in a shining leaf chafer
Source: Front Zool. 2025 Aug 4;22:18. doi: 10.1186/s12983-025-00571-5 (PMC12323268; doi:10.1186/s12983-025-00571-5)
Supplement: Supplementary file 1 — Additional file 1 [file 12983_2025_571_MOESM1_ESM.docx]

**Structural background of intraspecific color polymorphism and the driver of geographic patterns in a shining leaf chafer**

Yuanyuan Lu^1, 2^*, Alexander Kovalev^2^, Lulu Li^1, 3^, Chuchu Li^2^, Xinyi Zhu^1, 3^, Min He^1^, Xingke Yang^1^, Ming Bai^1, 3, 4^*, Stanislav N. Gorb^2^

**Supplementary materials**

**Additional file 1**

**Figure S1**. *Popillia mutans* in its natural environment.

**Table S1**. Selected environmental variables in this study.

**Table S2**. The selected environmental variables analyzed by Kruskal-Wallis One Way ANOVA on ranks.

**Additional file 2** (.xlsx file). The related analysis of color phenotype distribution pattern.

**Figure S2**. Violin plots showing the differences in the temperature related variables (Bio_2 to Bio_11) among different color phenotypes’ distribution.

**Figure S3**. Violin plots showing the differences in the precipitation related variables (Bio_13 to Bio_19) among different color phenotypes’ distribution.

**Figure S4**. Violin plots showing the differences in the monthly solar radiation (January to December, Srad_1 to 12) among different color phenotypes’ distribution.

**Figure S5**. Violin plots showing the differences in the monthly mean temperature (January to December, Tavg_1 to 12) among different color phenotypes’ distribution.

**Additional file 3** (.xlsx file). Measured and simulated reflectivity.

**Figure S6**. The SEM images of the dorsal surface of elytra in different color phenotypes.

**Additional file 4** (.xlsx file). Nanoindentation result.

**Additional file 5** (.xlsx file). Thermoregulation result.


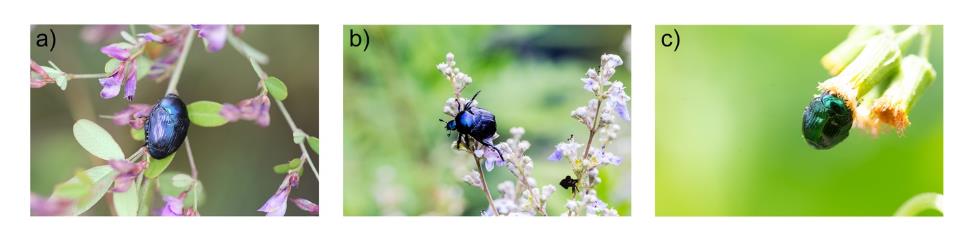


**Figure S1**. *Popillia mutans* in its natural environment. a) Blue: Beijing, China (Photographed by Chunyan Jiang). b) Blue: Hebei, China (Photographed by Yiping Niu). c) Green, Zhejiang, China (Photographed by Yandong Chen).

**Table S1**. Selected environmental variables in this study.

| **Variables** | **Variables description [Unit]** |
| --- | --- |
| Bio_1 | Annual mean temperature [℃] |
| Bio_2 | Mean diurnal temperature range (mean of monthly (max temp−min temp)) [℃] |
| Bio_3 | Isothermality (Bio_2/Bio_7) (×100) [/] |
| Bio_4 | Temperature seasonality (standard deviation×100) [/] |
| Bio_5 | Max temperature of the warmest month [℃] |
| Bio_6 | Min temperature of coldest month [℃] |
| Bio_7 | Temperature annual range (Bio_5−Bio_6) [℃] |
| Bio_8 | Mean temperature of the wettest quarter [℃] |
| Bio_9 | Mean temperature of the driest quarter [℃] |
| Bio10 | Mean temperature of warmest quarter [℃] |
| Bio11 | Mean temperature of the coldest quarter [℃] |
| Bio12 | Annual precipitation [mm] |
| Bio13 | Precipitation of wettest month [mm] |
| Bio14 | Precipitation of driest month [mm] |
| Bio15 | Precipitation seasonality (coefficient of variation) [/] |
| Bio16 | Precipitation of wettest quarter [mm] |
| Bio17 | Precipitation of driest quarter [mm] |
| Bio18 | Precipitation of the warmest quarter [mm] |
| Bio19 | Precipitation of the coldest quarter [mm] |
| Tavg_1 to 12 | Monthly mean temperature January to December [℃] |
| Srad_1 to 12 | Monthly solar radiation January to December [kJ m^-2^ day^-1^] |

**Table S2**. The selected environmental variables analyzed by Kruskal-Wallis One Way ANOVA on ranks.

| Variables | *p* value | Parameter (d.f.=3) |
| --- | --- | --- |
| Latitude | ****p*<0.001 | H=231.355 |
| Longitude | NS. *p*> 0.05 (0.169) | H=5.039 |
| Altitude | ****p*<0.001 | H=38.000 |
|  |  |  |
| Bio_1 | ****p* <0.001 | H=236.868 |
| Bio_12 | ****p* <0.001 | H=183.67 |
| Bio_2 | ****p* <0.001 | H=127.624 |
| Bio_3 | ****p* <0.001 | H=46.363 |
| Bio_5 | ****p* <0.001 | H=99.819 |
| Bio_8 | ****p* <0.001 | H=16.618 |
| Bio_15 | ****p* <0.001 | H=112.300 |
| Bio_18 | ****p* <0.001 | H=126.439 |
| Srad_6 | ****p* <0.001 | H=114.376 |
| Srad_7 | * *p* <0.05 (0.019) | H=9.969 |
| Srad_8 | ****p* <0.001 | H=36.359 |
| Tavg_6 | ****p* <0.001 | H=141.08 |
| Tavg_7 | ****p* <0.001 | H=140.169 |
| Tavg_8 | ****p* <0.001 | H=162.608 |

**Additional file 2** (.xlsx file). The related analysis of color phenotype distribution pattern. a. 544 specimens: synthesizing data on the geographic distributions of *Popillia mutans*'s colour phenotypes. b. Clim information: environmental data extracted from each specimen locality. c. PCA analysis: importance of each principal component and correlation of each climate variable with each principal component. d. Pearson correlation analysis: Pearson’s pairwise correlations between independent variables.


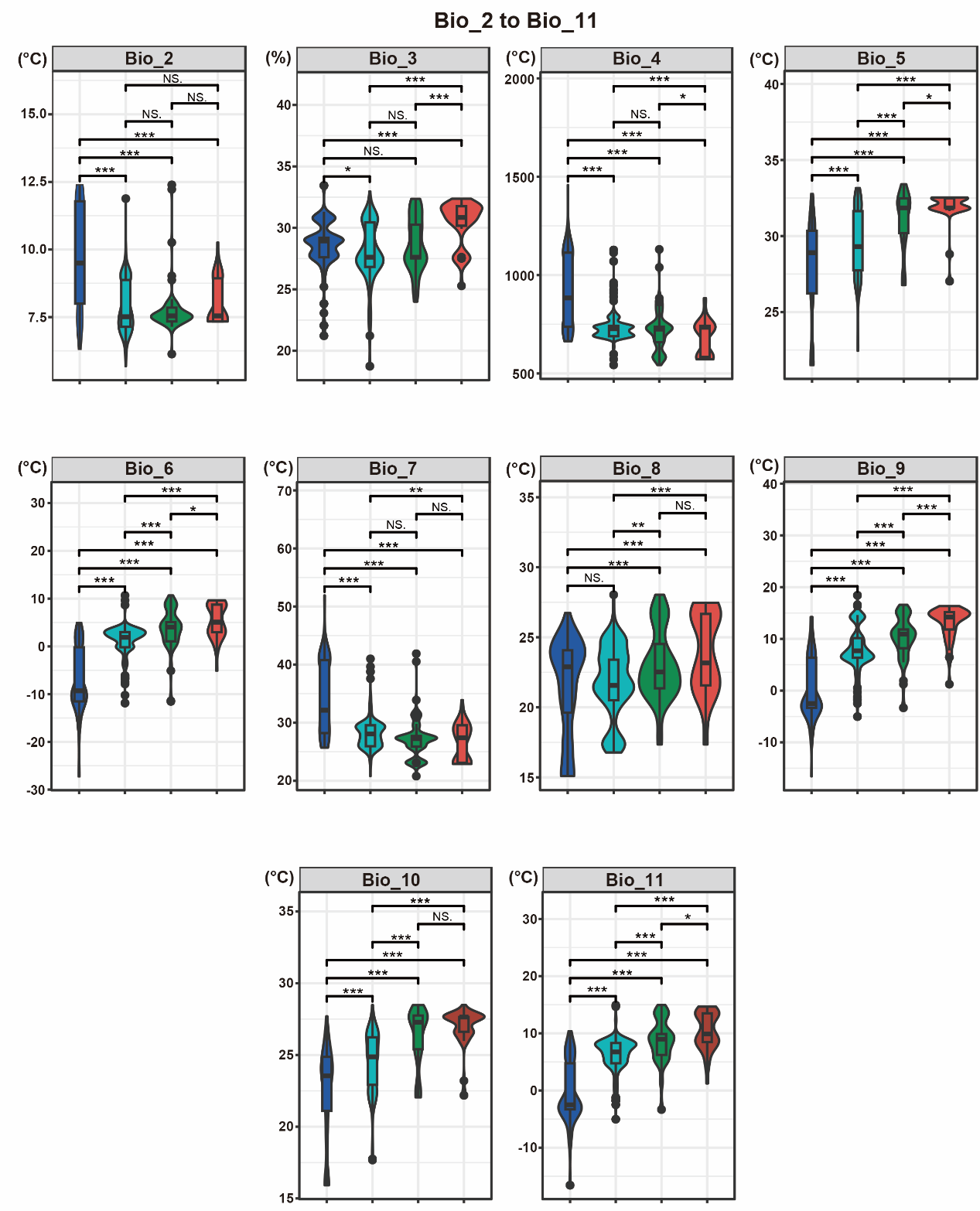


**Figure S2**. Violin plots showing the differences in the temperature related variables (Bio_2 to Bio_11) among different color phenotypes’ distribution. Bio_2 to Bio_11 see Table S1. Significant differences were evaluated by the *t*-test. Asterisks indicate the level of significance (NS *p*> 0.05, **p*< 0.05, ***p*< 0.01 and ****p*< 0.001).


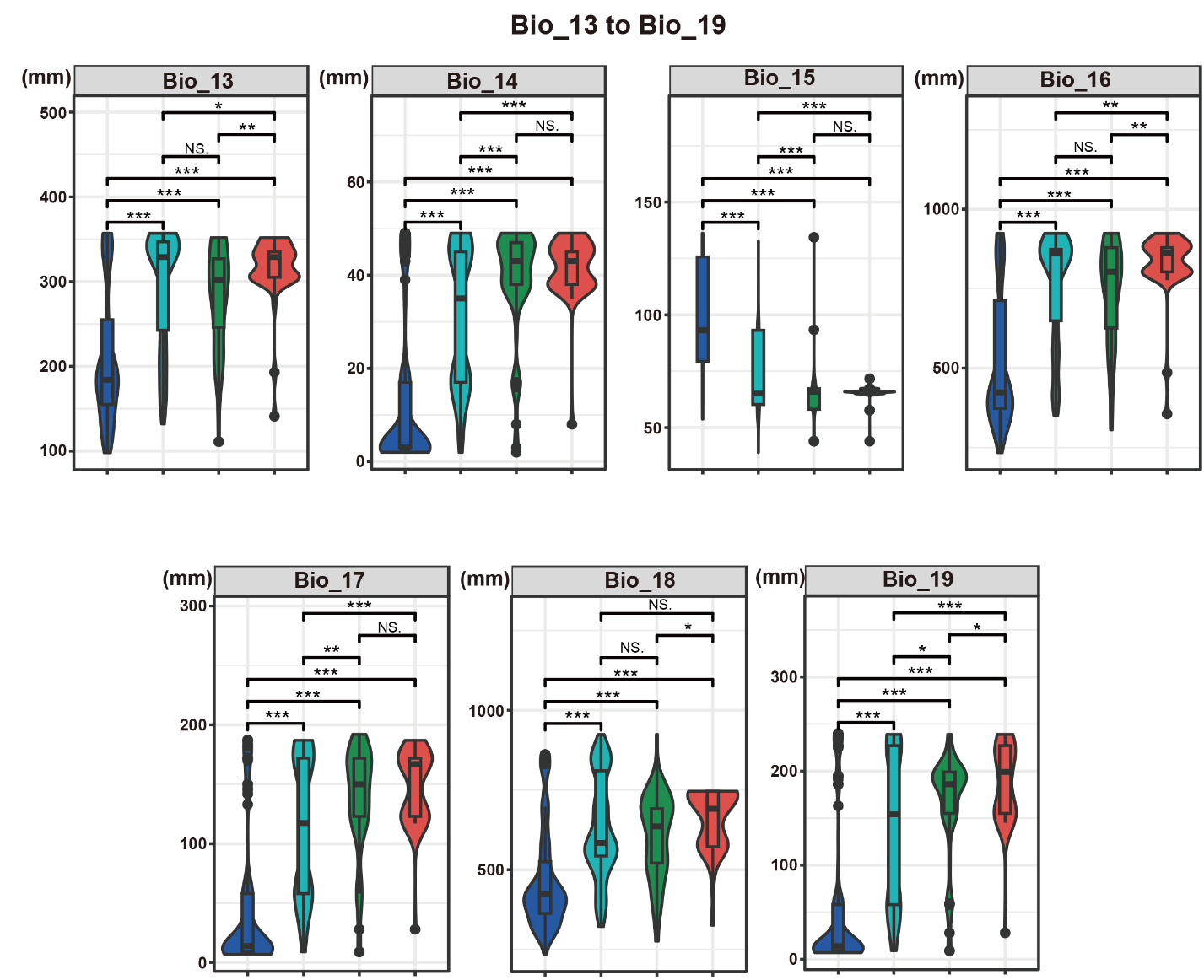


**Figure S3**. Violin plots showing the differences in the precipitation related variables (Bio_13 to Bio_19) among different color phenotypes’ distribution. Bio_13 to Bio_19 see Table S1. Significant differences were evaluated by the *t*-test. Asterisks indicate the level of significance (NS *p*> 0.05, **p*< 0.05, ***p*< 0.01 and ****p*< 0.001).


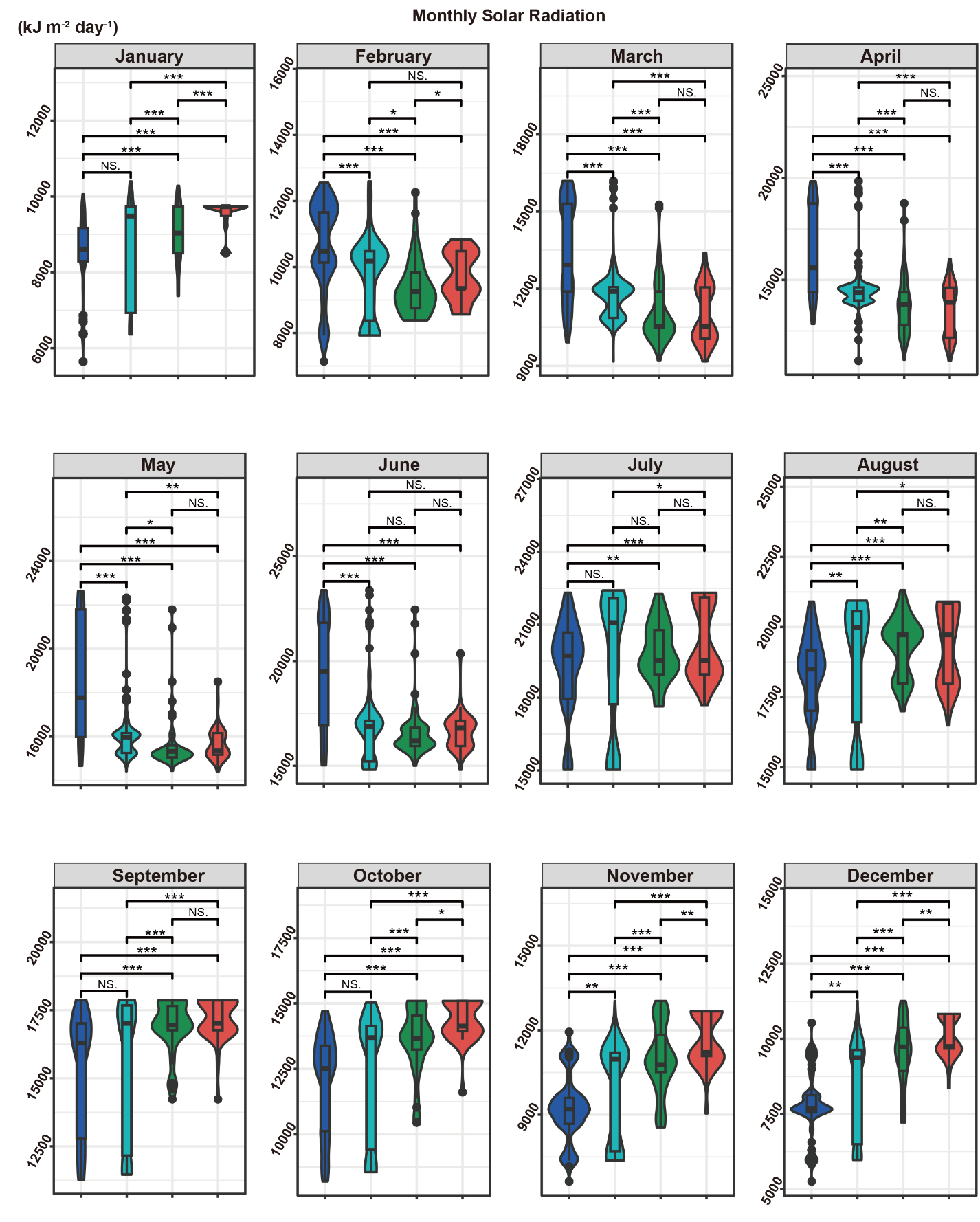


**Figure S4**. Violin plots showing the differences in the monthly solar radiation (January to December, Srad_1 to 12) among different color phenotypes’ distribution. Srad_1 to Srad_12 see Table S1. Significant differences were evaluated by the *t*-test. Asterisks indicate the level of significance (NS *p*> 0.05, **p*< 0.05, ***p*< 0.01 and ****p*< 0.001).


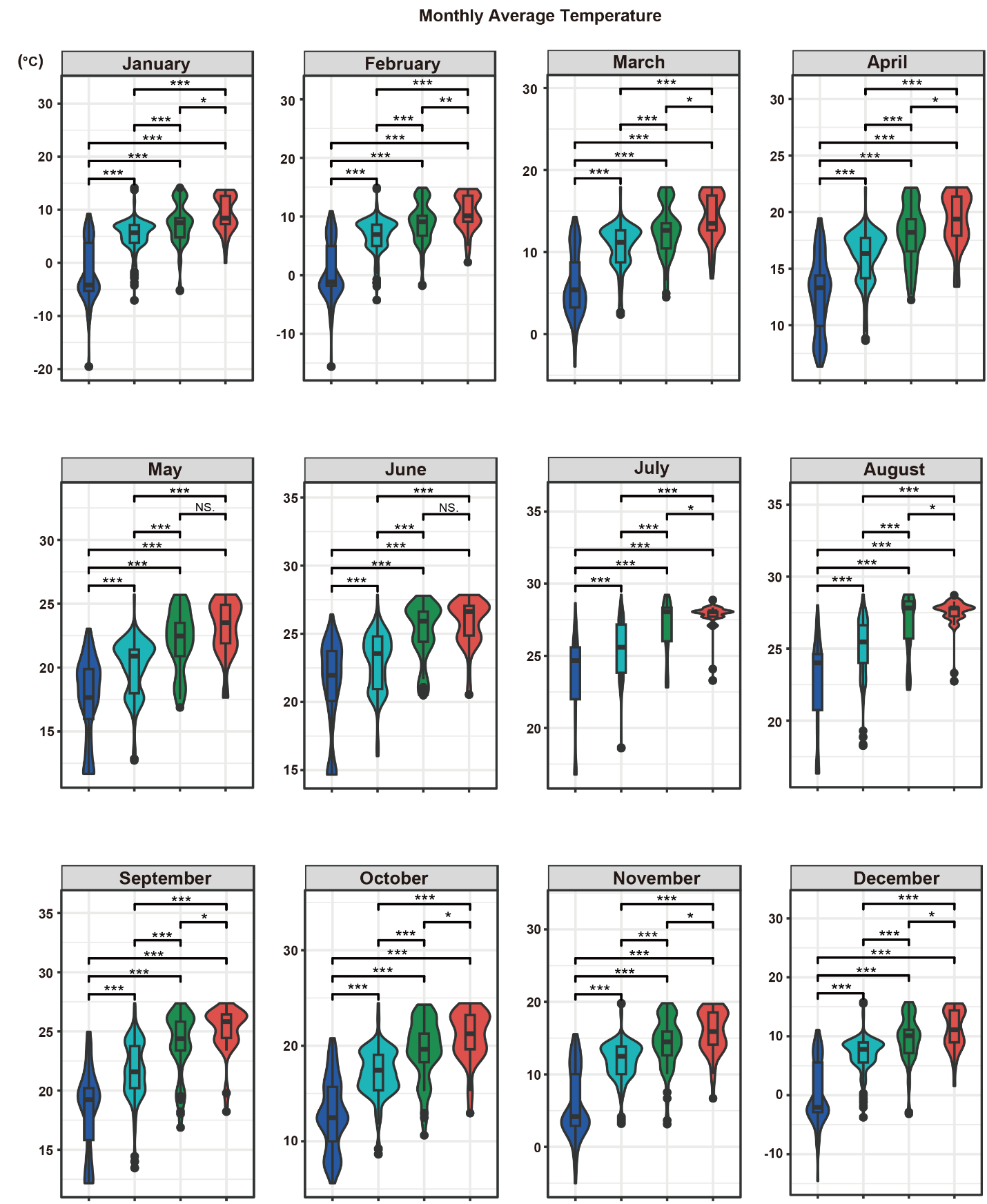


**Figure S5**. Violin plots showing the differences in the monthly mean temperature (January to December, Tavg_1 to 12) among different color phenotypes’ distribution. Tavg_1 to Tavg_12 see Table S1. Significant differences were evaluated by the *t*-test. Asterisks indicate the level of significance (NS *p*> 0.05, **p*< 0.05, ***p*< 0.01 and ****p*< 0.001).

**Additional file 3** (.xlsx file). Measured and simulated reflectivity. a, Sample information: the specimens' used in the reflectivity test. b, Reflectivity result: reflectivity measured from 380 nm to 780 nm. c, Elytral thickness used in simulation of reflectivity.


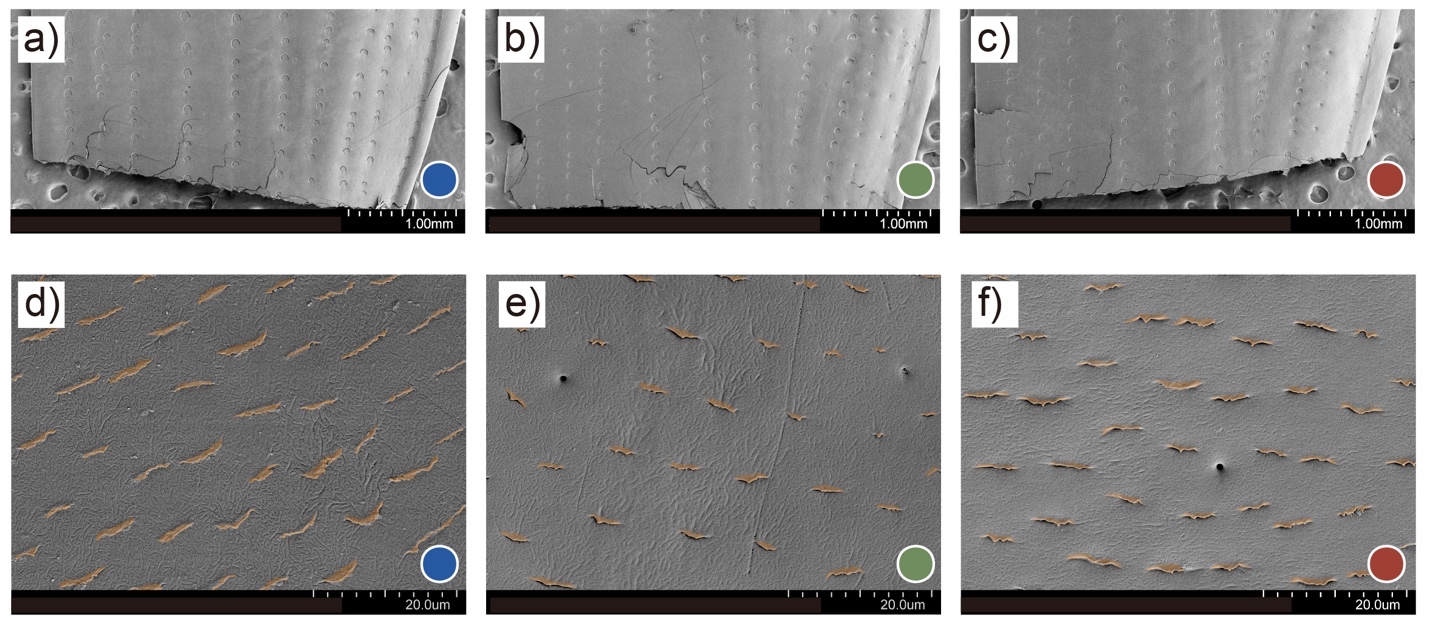


**Figure S6**. The SEM images of the dorsal surface of elytra in different color phenotypes. a-c, overview of the middle part in elytra, show lined punctures and smooth surface. d-f, ridges in smooth area, ridges are colored yellow. a, d, blue color phenotype; b, e, green color phenotype; d, f, red color phenotype.

**Additional file 4** (.xlsx file). Nanoindentation result. a, Sample information: the specimens’ used in the nanoindentation test. b, Indentation results: elasticity modulus and hardness measured in each indent. c, Sample results: the results of average modulus and hardness measured in each sample. d, Statistical analysis: one way ANOVA and *t*-test results of elasticity modulus and hardness between color phenotypes.

**Additional file 5** (.xlsx file). Thermoregulation result. a, Sample information: the specimens’ used in the thermoregulation test. b, Original results: the results of average temperature measured in each experiment. c, Statistical analysis.
